# Association Between Diet Diversity and Sleep Quality Among Medical Students: A Cross‐Sectional Study at Rawalpindi Medical University

**DOI:** 10.1002/hsr2.72745

**Published:** 2026-06-30

**Authors:** Zainab Salam, Muddassir Khalid, Sobia Safeer, Hunzla Saleem, Rehab Khalid, Muhammad Ahmad Bin Kashif, Laiba Yumn, Syed Farooq Shafiq, Hadia Jamil, Sumia Fatima, Fred Segawa

**Affiliations:** ^1^ Nawaz Sharif Medical College Gujrat Pakistan; ^2^ Nishtar Medical University Multan Pakistan; ^3^ Niazi Medical and Dental College Sargodha Pakistan; ^4^ Quaid‐e‐Azam Medical College Bahawalpur Pakistan; ^5^ Fazaia Medical College Islamabad Pakistan; ^6^ Khyber Medical College Peshawar Pakistan; ^7^ Rawalpindi Medical University Rawalpindi Pakistan; ^8^ Makerere University College of Health Sciences Kampala Uganda

**Keywords:** diet (D004032), medical (D013337), Pakistan (D010154), sleep (D012890), sleep quality (D000089943), students

## Abstract

**Introduction:**

Sleep represents a fundamental pillar of human health. Despite growing evidence highlighting its association with diet, limited data exist on how overall diet diversity relates to sleep health among medical students in low‐ and middle‐income countries.

**Objective:**

This study aims to assess the association between diet diversity and sleep quality among medical students.

**Methodology:**

A cross‐sectional study was conducted at Rawalpindi Medical University among undergraduate medical students from first to final year. A sample size of 246 participants was selected using non‐probability purposive sampling. Data were collected using the Diet Quality Questionnaire (DQQ), from which the Dietary Diversity Score (DDS) was derived, along with the Pittsburgh Sleep Quality Index (PSQI) and Healthy Eating Index (HEI). Data were analyzed using IBM SPSS Statistics version 26. Poor sleep quality was defined as a PSQI global score > 5.

**Results:**

A total of 246 undergraduate medical students participated in the study, of which 61% were female. More than 70% of participants reported poor sleep quality. Sleep quality showed no significant differences across gender (*p* = 0.9), year of study, or resident status. Higher body mass index was significantly associated with poorer sleep (*p* = 0.04). Higher diet diversity was associated with better sleep quality, with each one‐point increase in diet diversity score associated with an 11.8% reduction in the odds of poor sleep (B = −0.126, *OR* = 0.88, 95% CI 0.78–1.00, *p* = 0.042). Students with good sleep had higher median diet diversity scores compared with those with poor sleep (Mann–Whitney *U* = 5312.5, Z = −1.984, *p* = 0.047). Sleep medication use was significantly higher among females (*p* < 0.001).

**Conclusion:**

A high prevalence of poor sleep quality (> 70%) was observed among both male and female medical students. Most notably, greater diet diversity was associated with better sleep quality. Improving diet diversity and managing BMI could be strategic targets for enhancing sleep quality among medical students.

## 1 Introduction

Sleep is an important lifestyle factor that influences various metabolic and physiological processes, including the functioning of the cardiovascular and immune systems, as well as neurological and psychomotor activities. Emerging evidence suggests that dietary diversity influences sleep regulation through inflammatory, metabolic, and circadian pathways [1]. Sleep accounts for one‐third of the lifetime of an individual, with a duration of about 7–8 h daily for most age groups, as recommended by the National Sleep Foundation [2]. Numerous studies have shown a strong link between poor sleep quality and multimorbidities, such as cognitive dysfunction, cardiovascular diseases, diabetes, obesity, and depression. Sound sleep is important in maintaining human homeostasis and promoting health. However, many aspects of modern life, such as staying awake at night and increased screen time, can disrupt both the quality and duration of sleep [3].

The relationship between sleep and diet diversity has become a significant area of interest in public health and clinical studies because of its implications for both mental and physical health. Sleep and nutrition are two fundamental cornerstones of health, yet their complex two‐way interaction stays underexplored in many populations. Poor diet, a leading risk factor for various health‐related problems, is linked to poor sleep outcomes [4].

Sleep deprivation is common among university and medical students. University and medical students are notorious for their poor sleeping habits. Medical students worldwide have reported decreased sleep duration, insomnia, and increased daytime sleepiness [5]. Sixty‐three percent of American college students experience sleep disturbances, irregular sleeping patterns, and poor sleep quality. It was hypothesized that the quality of diet can positively or negatively influence sleep quality depending on dietary composition and diversity [6]. As lifestyles become more hectic and the consumption of processed food rises worldwide, understanding how diet diversity affects sleep has become extremely crucial. The purpose of our research is to investigate the association of diet diversity with sleep quality among medical students.

The study hypothesized that those with short sleep (less than 7 h per night) and long sleep (more than 9 h per night) would have poor diet quality, that is, lower adherence to the Mediterranean diet. Notably, both sleep and diet are modifiable factors that have a great impact on the risk of cardiovascular diseases [7]. According to a study conducted in Islamabad, Pakistan. About 41.14% of students have inadequate sleep even on non‐exam days [8]. Researchers estimated the prevalence of sleeping problems among university students to be between 15% and 70% [9]. According to a study about unhealthy dietary habits, 58% of students ate two meals per day; most of them skipped breakfast. The majority of the students (46%) consumed junk foods 1–2 times per week [10].

Recognizing that health‐related behaviors often intersect, the study focuses on the examination of nutritional knowledge as a potential influence on various health habits, particularly its impact on the quality of sleep among medical students [11]. The term “diet quality” has become popular over the past 2 decades in the scientific literature. Insufficient sleep was associated with poor diet and eating habits [12, 13]. This study investigates the bidirectional relationship between sleep quality and dietary diversity among medical students, exploring how food choices affect sleep and vice versa. Diet diversity may influence sleep through several biological mechanisms, including micronutrient availability for neurotransmitter synthesis (e.g., serotonin, melatonin), modulation of systemic inflammation, and effects on glycaemic control and circadian rhythms.

## 2 Materials and Methods

A cross‐sectional study design was employed to evaluate the association between diet diversity and sleep quality among medical students. The study population comprised all male and female medical students enrolled in the MBBS program, ranging from first‐year students to final‐year students, aged between 19 and 25 years. Data were collected using non‐probability purposive sampling.

## 3 Study Setting and Place

The study was conducted in May 2025 at Rawalpindi Medical University. The total study duration was approximately 2 months. Each participant was provided with an overview of the study's objectives, and confidentiality and anonymity of responses were ensured.

## 4 Sample Size

Sample size was calculated assuming a 95% confidence level and 80% power, based on effect estimates from prior literature [14]. The standard Cochrane formula for proportions was used:

$$n=Z^{2}\times p\left(1–p\right)/d^{2}$$

Assuming an expected prevalence (*p*) of 0.20 based on previous studies reporting poor sleep quality among medical students in Pakistan, where prevalence has been reported to be approximately 39.5% [14], a conservative estimate was used to ensure an adequate sample size. With a 95% confidence level (Z = 1.96) and a margin of error of 5% (d = 0.05), the minimum required sample size was calculated to be 246 participants.

## 5 Participants

### 5.1 Inclusion Criteria

Medical students aged 19–25 years enrolled in the MBBS program from first year through final year.

### 5.2 Exclusion Criteria

Students with diagnosed sleep disorders, chronic medical conditions, pregnancy, eating disorders, or those using medications known to affect sleep were excluded.

### 5.3 Ethical Considerations

All procedures were conducted in accordance with relevant guidelines and regulations. The study adhered to the Declaration of Helsinki. Ethical approval was obtained from the Ethics Committee of Rawalpindi Medical University, Rawalpindi, Pakistan (Ethical code: PH‐04‐05) on November 6, 2024. Informed consent was obtained from all participants prior to data collection.

### 5.4 Data Collection

Data were collected using a self‐administered, paper‐based questionnaire comprising the Diet Quality Questionnaire (DQQ) and the Pittsburgh Sleep Quality Index (PSQI). Dietary data were obtained through the Diet Quality Questionnaire (DQQ), from which the Dietary Diversity Score (DDS) was derived. The Healthy Eating Index (HEI) was used to assess overall diet quality. Sleep quality was assessed using the Pittsburgh Sleep Quality Index (PSQI). Anthropometric data, including height and weight, were collected through a structured self‐administered questionnaire. Participants were asked to report their height and weight, which were used to calculate body mass index (BMI) using the formula weight (kg)/height (m^2^).

### 5.5 Study Tools

The Pittsburgh Sleep Quality Index (PSQI) questionnaire was used to assess sleep quality, including components such as sleep duration, sleep disturbances, sleep latency, sleep efficiency, and use of sleep medication. Dietary assessment was conducted using the Diet Quality Questionnaire (DQQ), which served as the primary instrument for collecting data on food consumption patterns. The Dietary Diversity Score (DDS) was derived from responses obtained through the DQQ to assess dietary diversity, while the Healthy Eating Index (HEI) was used to evaluate overall diet quality. Together, these tools provided a comprehensive assessment of dietary diversity, diet quality, and sleep quality among participants.

### 5.6 Data Analysis

Data were analyzed using IBM SPSS Statistics version 26 (IBM Corp., Armonk, NY, USA). Descriptive statistics were presented as frequencies and percentages. The chi‐square test or Fisher's exact test was used to assess associations between categorical variables. A *p*‐value of < 0.05 was considered statistically significant.

## 6 Results

The study included 246 undergraduate medical students, aged 19–25 years, participated in the study. Female students constituted 61.4% (*n* = 151) of the sample, while males accounted for 38.6% (*n* = 95). Poor sleep quality (PSQI global score > 5) was reported by 70.2% (*n* = 106) of female participants and 70.5% (*n* = 67) of male participants. There was no significant relationship between gender and sleep quality (*p* = 0.9, *a* = 0.05) (Table 2).

There was no statistically significant association between year of study or resident status and sleep quality (*p* > 0.05) (Table 1).

**TABLE 1:** Sociodemographic and anthropometric characteristics of study participants (*n* = 246).

Variables	Overall (*n* = 246)	Participants with good sleep quality (*n* = 73)	Participants with Poor sleep quality (*n* = 173)
	*n* (%)	*n* (%)	*n* (%)
Gender			
Male	95 (38.6)	28 (29.5)	67 (70.5)
Female	151 (61.4)	45 (29.8)	106 (70.2)
Resident status			
Day scholars	70 (28.5)	25 (35.7)	45 (64.3)
Hostelers	176 (71.5)	48 (27.3)	128 (72.7)
Year of study			
1st year	40 (16.3)	10 (25.0)	30 (75.0)
2nd year	16 (6.5)	4 (25.0)	12 (75.0)
3rd year	34 (13.8)	8 (23.5)	26 (76.5)
4th year	58 (23.6)	12 (20.7)	46 (79.3)
Final year	98 (39.8)	39 (39.8)	59 (60.2)
BMI category			
Underweight (< 18.5)	38 (15.4)	9 (23.7)	29 (76.3)
Normal (18.5–24.9)	169 (68.7)	59 (34.9)	110 (65.1)
Overweight (25–29.9)	30 (12.2)	4 (13.3)	26 (86.7)
Obese (≥ 30)	9 (3.7)	1 (11.1)	8 (88.9)

**TABLE 2:** Sleep parameters based on Pittsburgh Sleep Quality Index (PSQI).

Sleep parameters	Most common response	Percentage (%)	Notable findings
Sleep duration	7–8 h	72	< 5% slept < 4 h
Insomnia symptoms	None in past month	40–80	20%–60% reported occasional symptoms
Sleep medication use	None	87.4	Rare daily use (< 6.1%)
Daytime sleepiness	None	50	30% experienced weekly episodes
Task enthusiasm	Fairly good	53.3	31.7% reported some difficulty
Bed partner/roommate	Partner in same room (different bed)	45.5	40.2% had no partner
Snoring	None	81.7	13% reported loud snoring weekly
Apnea episodes	None	85.8	Rare occurrence
Leg twitching	None	68.7	16.7% reported presence
Confusion during sleep	None	71.5	17.9% reported episodes
Other restlessness	None	70.7	17.5% reported symptoms

There is a significant relationship of BMI with sleep quality (*p* = 0.04).

There is a strong relationship between gender and use of sleep medications (prescribed or OTC drugs) (*p* < 0.001). 92.7% of females (*n* = 140) were using sleep medications to get adequate sleep. In contrast, 78.9% of males (*n* = 75) reported using sleep medications. There was no significant relationship between gender, sleep duration, and sleep efficiency.

There is no significant relationship of resident status with use of sleep medications (*p* = 0.1, *a* = 0.05). There was no significant correlation found between the resident status and the duration and efficiency of sleep (Tables 3 and 4).

**TABLE 3:** Dietary diversity score (DDS) and healthy eating index (HEI).

Measure	Mean ± SD	Min–Max
Dietary Diversity Score (DDS)	6.2 ± 1.4	3–10
Healthy Eating Index (HEI)	56.8 ± 9.7	34–82

**TABLE 4:** Dietary scores by sleep quality status.

Variable	Good sleep (*n* = 99)	Poor sleep (*n* = 146)	*P*
DDS	6.8 ± 1.3	5.9 ± 1.4	0.003
HEI	60.9 ± 9.2	54.3 ± 9.4	< 0.001

### 6.1 Binary Logistic Regression

A binary logistic regression was conducted to determine the association between diet diversity score (DDS) and sleep quality status (PTSQI). The overall model was statistically significant, χ^2^ (1) = 4.179, *p* = 0.041, indicating that DDS significantly contributed to the prediction of poor sleep quality.

The model explained 1.7%–2.4% of the variance (Cox & Snell *R* ^2^ = 0.017, Nagelkerke *R* ^2^ = 0.024) and demonstrated a satisfactory fit to the data (Hosmer–Lemeshow test: *p* = 0.977).

DDS was found to be a significant negative predictor of poor sleep (B = –0.126, SE = 0.062, *p* = 0.042). The odds ratio (Exp(B) = 0.882, 95% CI [0.781, 0.996]) suggests that each one‐unit increase in DDS was associated with an 11.8% decrease in the odds of reporting poor sleep. Dietary Diversity Score was identified as a significant independent negative predictor of poor sleep quality (B = −0.126, SE = 0.062, *OR* = 0.882, 95% CI: 0.781–0.996, *p* = 0.042), indicating that each one‐unit increase in DDS was associated with an 11.8% reduction in the odds of poor sleep. The overall model was statistically significant (χ^2^ (1) = 4.179, *p* = 0.041), explaining 1.7%–2.4% of the variance and demonstrating good calibration (Hosmer–Lemeshow *p* = 0.977).

### 6.2 Mann–Whitney *U* Test

To further determine the association between diet diversity score and sleep quality, a Mann–Whitney *U* test was conducted to compare DDS scores between participants with good and poor sleep quality. Results showed a statistically significant difference in DDS scores between the two groups, U = 5312.5, Z = –1.984, *p* = 0.047.

Participants who reported good sleep quality (*n* = 73) had higher DDS scores (mean rank = 137.23) compared to those who reported poor sleep quality (*n* = 173; mean rank = 117.71). This indicates that individuals with better sleep quality tend to consume a more diverse diet.

These findings support the binary logistic regression results, suggesting that higher diet diversity is associated with better sleep quality.

## 7 Discussion

This cross‐sectional study investigated the association between diet diversity scores and sleep quality among medical students, revealing several important findings that add to the body of literature on sleep health determinants in young adults.

Our results showed that more than 70% of both males and females reported poor sleep quality, and no significant gender difference was found in sleep quality status (*p* = 0.9). This contrasts with some previous studies indicating higher prevalence of poor sleep quality in females. For example, Fatima et al. (2016) reported significantly higher odds of poor sleep quality in young females; however, they suggested that such differences may be partly attributed to biological differences in sleep physiology rather than psychosocial factors alone [15]. The lack of difference in our study could be reflective of a relatively homogeneous population, such as medical students facing similar academic and lifestyle stressors. Our finding of a strong gender difference in sleep medication use, with females more likely to use sleep medications, aligns with other evidence showing gender disparities in sleep‐related health behaviors despite similar sleep quality reports [15].

No significant associations between years of study or resident status and sleep quality were observed, suggesting that academic seniority or living arrangements (day scholar vs. hostellite) do not considerably influence sleep quality among this population. This finding is consistent with earlier studies indicating that these demographic factors may not have a direct influence on sleep health compared to lifestyle and physiological factors.

A notable finding was the significant association between BMI and sleep quality (*p* = 0.04), where higher BMI, particularly overweight and obesity categories, was linked with poorer sleep quality. This echoes the extensive literature pointing to overweight and obesity as major risk factors for sleep disturbances, including sleep apnea and poor sleep efficiency, due to physiological mechanisms such as airway obstruction and systemic inflammation [16, 17]. The distribution of BMI and the pronounced detrimental effect of obesity on sleep reinforce the importance of weight management in promoting healthy sleep [18].

With respect to diet, we found that diet diversity score (DDS) was a significant negative predictor of poor sleep quality, with an 11.8% reduction in odds of poor sleep per unit increase in DDS. Participants with better sleep quality had higher DDS scores, indicating that a more varied diet is associated with improved sleep. This finding supports previous data suggesting that nutritional quality, including micronutrient diversity and consumption of healthy food groups, influences sleep regulation through pathways involving neurotransmitter synthesis, circadian rhythms, and inflammation modulation [19, 20]. The logistic regression and Mann–Whitney *U* test collectively reinforce the protective role of diet diversity in sleep health.

Regarding sleep patterns and quality, our findings highlight that a substantial portion of students experience delayed sleep latency (> 30 min in 41.5%), late bedtimes (35.4% sleeping at 2 a.m.), episodes of daytime sleepiness (30% weekly), and sporadic insomnia symptoms. While most participants did not frequently use sleep medications or report apnea, mild sleep disturbances such as leg twitching, confusion, and other restlessness were reported by roughly 15%–18% of participants. These observations are aligned with typical stress‐related sleep disturbances in medical students, who are known to experience academic pressures and irregular lifestyles compromising sleep [21, 22].

Taken together, our results underscore the multifactorial nature of sleep quality determinants in medical students, where physiological (BMI), behavioral (diet), and demographic factors interact to shape sleep outcomes [23]. The absence of clear gender differences in sleep quality but pronounced differences in medication use suggest targeted interventions may be needed to address sleep management behaviors alongside broader lifestyle modifications [15, 24]. Despite these insights, several limitations warrant consideration. The cross‐sectional nature of this study prevents the establishment of temporal or causal relationships between diet quality, BMI, and sleep disturbances [25]. Furthermore, the reliance on self‐reported measures for both dietary intake and sleep quality may introduce recall bias or social desirability bias. Future research should utilize longitudinal designs and objective assessments, such as actigraphy or polysomnography, to better elucidate the physiological mechanisms linking nutritional variety to sleep architecture [26, 27]. Nevertheless, our findings suggest that medical institutions should prioritize holistic wellness programs that integrate nutritional counseling and weight management strategies to mitigate the high prevalence of poor sleep among students [28]. Promoting a diverse diet could serve as a non‐pharmacological, accessible tool for improving sleep health in this high‐stress population.

## 8 Limitations

Limitations of this study include its cross‐sectional design, which precludes causal inferences, reliance on self‐reported data subject to bias, and a sample limited to medical students, restricting generalizability. Future longitudinal studies examining diet, BMI, psychological stress, and objective sleep measures would provide greater understanding of causal pathways and mechanisms.

## 9 Conclusion

In conclusion, improving diet diversity and managing BMI could be strategic targets for enhancing sleep quality among medical students. We may also need to consider gender‐specific strategies for promoting sleep health, particularly those that target sleep medication use. These findings contribute to a growing body of evidence emphasizing holistic lifestyle approaches to improving sleep in young adult populations facing demanding academic environments.

## Author Contributions

**Zainab Salam:** conceptualization, methodology. **Muddassir Khalid:** supervision, writing – review and editing. **Sobia Safeer:** writing – original draft. **Hunzla Saleem:** writing – original draft. **Rehab Khalid:** software, data curation. **Muhammad Ahmad Bin Kashif:** data curation, visualization. **Laiba Yumn:** writing – review and editing. **Syed Farooq Shafiq:** writing – original draft. **Hadia Jamil:** writing – original draft. **Sumia Fatima:** writing – review and editing, validation. **Fred Segawa:** project administration.

## Funding

The authors have nothing to report.

## Ethics Statement

In this study, all methods were carried out in accordance with relevant guidelines and regulations. The study adhered to the Declaration of Helsinki in this regard. The current research was done after obtaining the approval of the Ethics Committee of Rawalpindi Medical University, Rawalpindi, Pakistan (Ethical code: PH‐04‐05) on November 6, 2024.

## Consent

Written informed consent from the subjects was obtained.

## Conflicts of Interest

The authors declare no conflicts of interest.

## Transparency Statement

The corresponding author affirms that this manuscript is an honest, accurate, and transparent account of the study being reported; that no important aspects of the study have been omitted; and that any discrepancies from the study as originally planned have been explained.

## Data Availability

The data that support the findings of this study are available from the corresponding author upon reasonable request.
